# Collectanea Medica

**Published:** 1813-12

**Authors:** 


### COLLECTANEA MEDICA,

CONSISTING OF

### ANECDOTES, FACTS, EXTRACTS, ILLUSTRATIONS, QUERIES, SUGGESTIONS, &c.

RELATING TO THE

*History or the Art of Medicine, and the Auxiliary Sciences.*

---

### *Biographical Account of M. de Fourcroy.* By THOMAS THOMSON, M.D. F.R.S.

LITERARY men may be divided into three classes. Some make a great figure during their life-time; but death erases their names from the annals of science, and they sink into the grave and obscurity at once. Such were Dr. Mead and Sir John Hill. Some are little known during their life-time, and spend their days in obscurity and penury; but when death has once closed the scene, their reputation rises untarnished by envy, and unsullied by emulation, and flows

 on on like a mighty river, the broader, and deeper, and greater, the farther it advances. Such, in some respects, were Kepler and Scheele. Some are so unfortunate, through imprudence, or a perverse train of circumstances, neither to acquire reputation during their lives, nor after their death; while their more successful contemporaries, with less labor, and less merit, gather all the laurels which they had earned. It would be invidious to mention the names of any who unfortunately belong to this class; but they will readily occur to every one acquainted with the history of science. Every tyro in algebra is familiar with Cardan's rules for the solution of cubic equations, while the name of the real discoverer of these rules is scarcely known, except to mathematical antiquaries. M. de Fourcroy, the subject of this article, made so conspicuous a figure during his life-time, that it would by no means surprise us if he should finally take his place among that class of literary men whom we characterised in the first place: not that he wanted merit; for it is not so much merit, as a regard to distributive justice, which leads to the classification. Who will be hardy enough to affirm that Churchill wanted merit as a poet? During his short and rapid literary career, he appeared to wield the thunderbolts in his hand, and was an object of dread and adoration, like a kind of divinity. But where is his reputation now? It has sunk, since his death, as much below the true level, as it rose above it during his life-time. And this we believe will always be the case. Mankind will atone for the excessive adulation which they pay to a man during his life-time, by a corresponding negligence after his death.

Antoine François de Fourcroy, Comte of the French Empire, Counsellor of State, Commander of the Legion of Honour, Member of the Institute, and of most scientific societies in Europe, Professor of Chemistry at the Museum of Natural History, Professor of the Faculty of Medicine at Paris, and Teacher in the Polytechnic School, was born at Paris, on the 15th of June, 1755, and was the son of Jean Michel de Fourcroy and of Jeanne Laugier.

His family had long resided in the capital, and several of his ancestors had distinguished themselves at the bar. One of them, during the reign of Charles IX. was honored with the epithet of *fori decus*.

Antoine François de Fourcroy sprung from a branch of the family that had gradually sunk into poverty. His father exercised in Paris the trade of an apothecary, in consequence of a charge which he held in the house of the Duke of Orleans. The Corporation of Apothecaries having obtained the general suppression of all such charges, M. de Fourcroy, the father, was was obliged to renounce his mode of livelihood; and his son grew up in the midst of the poverty produced by the monopoly of the privileged bodies in Paris. He felt this situation the more keenly, because he possessed from nature an extreme sensibility of temper. When he lost his mother, at the age of seven years, he attempted to throw himself into her grave. The care of an elder sister preserved him with difficulty till he reached the age at which it was usual to be sent to the college. Here he was unlucky enough to meet with a brutal master, who conceived an aversion to him, and treated him with cruelty. The consequence was a dislike to study; and he quitted the college at the age of 14, somewhat less informed than when he went to it.

His poverty now was such, that he was under the necessity of endeavoring to support himself by commencing writing-master. He had even some thoughts of going upon the stage; but was prevented by the hisses bestowed upon a friend of his, who had unadvisedly entered upon that perilous career, and was treated in consequence without mercy by the audience. While uncertain what plan to follow, the advice of Viq. d'Azyr induced him to commence the study of medicine.

This great anatomist was an acquaintance of M. de Fourcroy, the father. Struck with the appearance of his son, and the courage with which he struggled with his bad fortune, he conceived an affection for him, and promised to direct his studies, and even to assist him during their progress. The study of medicine to a man in his situation was by no means an easy task. He was obliged to lodge in a garret, so low in the roof that he could only stand upright in the centre of the room. Beside him lodged a water-carrier, with a family of twelve children. Fourcroy acted as physician to this numerous family; and in recompense was always supplied with abundance of water. He contrived to support himself by giving lessons to other students, by facilitating the researches of richer writers, and by some translations which he sold to a bookseller. For these he was only half paid; but the conscientious bookseller offered, thirty years afterwards, to make up the deficiency, when his creditor was become Director General of Public Instruction.

Fourcroy studied with so much zeal and ardour that he soon became well acquainted with the subject of medicine. But this was not sufficient. It was necessary to get a doctor's degree; and all the expenses, at that time, amounted to 250*l.* sterling. An old physician, Dr. Diest, had left funds to the faculty to give a gratuitous degree and license, once every two years, to the poor student who should best deserve them.

them. Fourcroy was the most conspicuous student at that time in Paris. He would therefore have reaped the benefit of this benevolent institution, had it not been for the unlucky situation in which he was placed. There happened to exist a quarrel between the faculty charged with the education of medical men and the granting of degrees, and a society recently establisbed by government for the improvement of the medical art. This dispute had been carried to a great length, and had attracted the attention of all the frivolous and idle inhabitants of Paris. Viq. d'Azyr was secretary to the society, and of course one of its most active champions, and was in consequence particularly obnoxious to the Faculty of Medicine at Paris. Fourcroy was unluckily the acknowledged protegée of this eminent anatomist. This was sufficient to induce the Faculty of Medicine to refuse him a gratuitous degree. He would have been excluded in consequence from entering upon the career of a practitioner, had not the society, enraged at this treatment, and influenced by a violent party spirit, formed a subscription, and contributed the necessary expences.

It was no longer possible to refuse M. de Fourcroy the degree of Doctor, when he was thus enabled to pay for it. But above the simple degree of Doctor, there was a higher one, entitled *Docteur Regent*, which depended entirely upon the votes of the faculty. It was unanimously refused to M. de Fourcroy. This refusal put it out of his power afterwards to commence teacher in the medical school, and gave the medical faculty the melancholy satisfaction of not being able to enrol among their number the most celebrated professor in Paris. This violent and unjust conduct of the faculty of medicine made a deep impression in the mind of Fourcroy, and contributed not a little to the subsequent downfall of that powerful body.

Fourcroy being thus entitled to practise in Paris, his success depended entirely upon the reputation which he could contrive to establish. For this purpose he devoted himself to the sciences connected with medicine, as the shortest and most certain road by which he could reach his object. His first writings showed no predilection for any particular branch of science. He wrote upon chemistry, anatomy, and on natural history. He published an *Abridgment of the History of Insects*, and a *Description of the Bursæ Mucosæ of the Tendons*. This last piece seems to have given him the greatest celebrity: for in 1785 he was admitted, in consequence of it, into the Academy of Sciences as an anatomist; but the reputation of Bucquet, which at that time was very high, gradually directed his particular attention to chemistry, chemistry, and he retained this predilection during the rest of his life.

Bucquet was at that time professor of chemistry in the medical school of Paris, and was then greatly celebrated and followed, on account of his eloquence and the elegance of his language. Fourcroy became in the first place his pupil, and soon after his particular friend. One day, when an unforeseen disease prevented him from lecturing as usual, he entreated M. de Fourcroy to supply his place. The young philosopher at first declined, and alleged his total ignorance of the method of addressing a popular audience. But, overcome by the persuasions of Bucquet, he at last consented; and in this his first essay, he spoke two hours without disorder or hesitation, and acquitted himself to the satisfaction of his whole audience. Bucquet soon after substituted him in his place, and it was in his laboratory and in his class-room that he first made himself acquainted with chemistry. He was enabled at the death of Bucquet, in consequence of an advantageous marriage which he had made, to purchase the apparatus and cabinet of his master; and although the Faculty of Medicine would not allow him to succeed to the chair of Bucquet, they could not prevent him from succeeding to his reputation.

There was a kind of college established in the King's Garden, which was at that time under the superintendance of Buffon, and Macquer was the professor of chemistry in this institution. On the death of this chemist, in 1784, Lavoisier stood candidate for the chair. But Buffon received more than a hundred letters in favor of Fourcroy; and the voice of the public was so loud in his favor, that he was appointed to the situation, in spite of the high reputation of his antagonist, and the superior interest that might be supposed to result from his fortune and his situation.

Fourcroy continued professor at the Jardin des Plantes during the remainder of his life, which lasted twenty-five years; and such was his eloquence, or so well was it fitted to the taste of the French nation, that his celebrity as a lecturer continued always upon the increase: so great also were the crowds, both of men and women, that flocked to hear him, that it was twice necessary to enlarge the size of the lecture-room. I had myself an opportunity of hearing him lecture two or three times, and must acknowledge that I found it difficult to account for the celebrity which he enjoyed. His style was precisely similar to that of his books, flowing and harmonious, but very diffuse, and destitute of precision; and his manner was that of a petit maitre, mixed with a good deal of pomposity, and an affectation of profundity, fundity. There must be something, however, in such a manner, capable of attracting the generality of mankind; for I know a professor who possesses as much of it as is consistent with the British character, and who is far inferior to Fourcroy as a man of science; who, nevertheless, enjoys within his own sphere nearly the same degree of popularity that Fourcroy did in his.

We must now notice the political career which Fourcroy ran during the progress of the revolution. In a country where political changes were going on with so much rapidity, and where every description of men were successively had recourse to, it was not possible that a professor so much admired for his eloquence could escape observation. Accordingly, he was elected a member of the National Convention in the autumn of 1793. The National Convention, and France herself, were at that time in a state of abject slavery; and so sanguinary was the tyrant who ruled over that unhappy country, that it was almost equally dangerous for the members of the Convention to remain silent, or to take an active part in the business of that assembly. Fourcroy, notwithstanding his reputation for eloquence, and the love of eclat which appears all along to have been his domineering passion, had good sense enough to resist the temptation, and never opened his mouth in the Convention till after the death of Robespierre. This is the more to be wondered at, and is a greater proof of prudence, as it is well known that he took a keen part in favor of the revolution, and that he was a determined enemy to the old order of things, from which he had suffered so severely at his entrance into life.

At this period he had influence enough to save the life of some men of merit: among others, of Darcet, who did not know the obligation he lay under to him till long after. At last his own life was threatened, and his influence of course utterly annihilated.

During this unfortunate and disgraceful period, several of the most eminent literary characters of France were destroyed; among others, Lavoisier, and Fourcroy has been accused of contributing to the death of this illustrious philosopher, his former rival, and his master in chemistry. How far such an accusation is deserving of credit, I have no means of determining; but Cuvier, who was upon the spot, and in a situation which enabled him to investigate its truth or falsehood, acquits Fourcroy entirely of the charge, and declares that it was urged against him merely out of envy at his subsequent elevation. "If, in the rigorous researches which we have made," says Cuvier, in his Eloge of Fourcroy, "we had found the smallest proof of an atrocity so horrible, no human power could have induced us to sully our mouths with his Eloge, or to have pronounced it within the walls of this temple, which ought to be no less sacred to honor than to genius.

Fourcroy began to acquire influence only after the 9th Thermidor, when the nation was wearied with destruction, and when efforts were making to restore those monuments of science, and those public institutions for education, which, during the wantonness and folly of the revolution, had been overturned and destroyed. Fourcroy was particularly active in this renovation, and it was to him chiefly that almost all the schools established in France for the education of youth are to be ascribed. The Convention had destroyed all the colleges, and universities, and academies, throughout France. The effects of this ridiculous abolition soon became visible. The army stood in need of surgeons and physicians, and there were none educated to supply the vacant places. Three new schools were founded for educating medical men. They were nobly endowed, and still continue connected with the University of Paris. The term *schools of medicine* was proscribed as too aristocratical. They were distinguished by the ridiculous appellation of *schools of health*. The *Polytechnic School* was next instituted, as a kind of preparation for the exercise of the military profession, where young men could be instructed in mathematics and natural philosophy, to make them fit for entering the schools of the artillery, of genius, and of the marine. The central schools was another institution for which France is indebted to the efforts of Fourcroy. The idea was good, though it has been very imperfectly put in execution. It was to establish a kind of university in every department, for which the young men were to be prepared by means of a sufficient number of inferior schools scattered through the department. But these inferior schools have never been either properly established or endowed; and even the central schools themselves have never been supplied with proper masters. Indeed it would have been impossible to have furnished such a number of masters at once. On that account an institution was established at Paris, under the name of *Normal School*, for the express purpose of educating a sufficient number of masters to supply the different central schools.

Fourcroy, either as member of the *Convention*, or of the *Council of Ancients*, took an active part in all these institutions, both as far as regarded the plan and the establishment. He was equally concerned in the establishment of the Institute, and of the *Museum d'Histoire Naturelle*. This last was endowed with the utmost liberality, and Fourcroy was one of the first professors; as he was, also, in the School of Medicine, and the Polytechnic School. He was equally concerned in the restoration of the University, which constitutes the most splendid part of Bonaparte's reign, and the part which will be longest remembered with gratitude and applause.

The violent exertions which M. de Fourcroy made in the numerous situations which he filled, and the prodigious activity which he displayed, gradually undermined his constitution. He himself was sensible of his approaching death, and announced it to his friends as an event which would speedily take place. On the 16th of December, 1809, after signing some dispatches, he suddenly cried out *Je suis mort*, and dropt lifeless on the ground.

He was twice married: first to Mademoiselle Bettinger, by whom he had two children; a son, an officer in the artillery, who inherits his title; and a daughter, Madame Foucaud. He was married a second time to Madame Bellville, the widow of Vailly, by whom he had no family. He left but little fortune behind him; and two maiden sisters who lived with him, depended, for their support, upon his friend M. Vauquelin.

(To be continued.)

